# Polarization-resolved spectroscopy imaging of grain boundaries and optical excitations in crystalline organic thin films

**DOI:** 10.1038/ncomms9201

**Published:** 2015-09-14

**Authors:** Z. Pan, N. Rawat, I. Cour, L. Manning, R. L. Headrick, M. Furis

**Affiliations:** 1 Department of Physics, Materials Science Program, University of Vermont, Burlington, Vermont 05405, USA

## Abstract

Exploration of optical properties of organic crystalline semiconductors thin films is challenging due to submicron grain sizes and the presence of numerous structural defects, disorder and grain boundaries. Here we report on the results of combined linear dichroism (LD)/ polarization-resolved photoluminescence (PL) scanning microscopy experiments that simultaneously probe the excitonic radiative recombination and the molecular ordering in solution-processed metal-free phthalocyanine crystalline thin films with macroscopic grain sizes. LD/PL images reveal the relative orientation of the singlet exciton transition dipoles at the grain boundaries and the presence of a localized electronic state that acts like a barrier for exciton diffusion across the grain boundary. We also show how this energy barrier can be entirely eliminated through the optimization of deposition parameters that results in films with large grain sizes and small-angle boundaries. These studies open an avenue for exploring the influence of long-range order on exciton diffusion and carrier transport.

## Abstract (web summary)

In-depth understanding of organic crystalline semiconductor thin films is critical for the development of many electronic and photonic devices. Here, the authors use combined linear dichroism and polarization-resolved photoluminescence scanning microscopy to unveil the nature of excited states in such systems.

## Graphical abstract

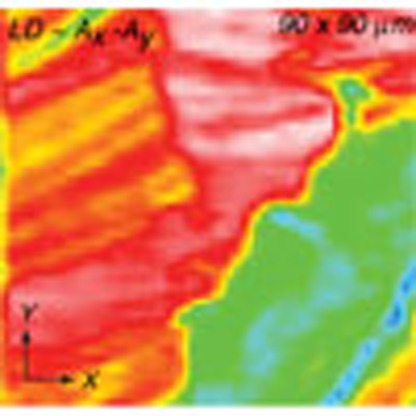

Research on organic semiconductors has advanced tremendously for the past two decades as these materials have great potential for the development of novel electronic and photonic devices^1^,^2^. Among them, small molecules with finite, well-defined *π*-conjugated systems, such as pentacene, rubrene, perylene or phthalocyanine, exhibit very large charge carrier mobilities^3^,^4^,^5^, and represent a cost-effective flexible electronics alternative for certain traditional silicon-based semiconductor applications such as field-effect transistors and photovoltaic devices^5^,^6^,^7^,^8^. While it is clear that the electronic properties of such films are highly tunable by chemical methods that simply modify their molecular building blocks or by physical methods through different fabrication techniques (chemical vapour deposition, spin coating, zone casting and molecular beam epitaxy)^5^,^6^, there is a critical need for a deeper fundamental understanding of the influence of long-range ordering on collective phenomena such as exciton diffusion and recombination, or carrier transport. X-ray, electron microscopy and other structural characterization techniques that provide feedback on the molecular packing and crystalline symmetry cannot selectively probe correlations between itinerant electrons.

Optical spectroscopy techniques, such as photoluminescence (PL), differential absorption and circular or linear dichroism (LD), are powerful investigation methods for collective excitations in solids^9^,^10^. However, in organic films they are severely limited by the large degree of disorder present on the length scale of optical microscopy techniques (∼1 μm). In many cases, the large concentration of defects effectively quenches the luminescence. For small-molecule polycrystalline films, it is very difficult to study correlations between long-range order and excitons because the typical grain sizes are only in the tens to hundreds of nanometres range and the recorded optical response contains contributions from many randomly oriented grains. Novel solution-based deposition methods^6^,^11^ that produce polycrystalline films with macroscopic millimetre-sized grains offer the opportunity for the first time to employ the wealth of powerful optical spectroscopy techniques to gain insight into the fundamental links between the electronic states, long-range order and the role of grain boundaries in crystalline thin films of organic semiconductors.

In this paper, we report on a spectrally resolved PL/LD polarization microscopy study of excitonic states in individual crystalline grains of metal-free phthalocyanine (H_2_Pc) films fabricated with a novel hollow capillary pen-writing deposition technique^11^,^12^,^13^. An in-house built laser scanning polarization-resolved microscopy setup records 90 × 90-μm-LD images, and probes the symmetry of optically allowed electronic states with a spatial resolution of ∼5 μm. These images reveal how the relative orientation of exciton dipoles at the grain boundaries can be controlled in a marked manner through fine tuning of the deposition parameters (that is, pen-writing speed and solution concentration). The same microscopy setup simultaneously records the polarization-resolved PL from single crystalline grains or individual grain boundaries, which can be correlated with the orientation of optically allowed dipoles. The PL spectra of grain boundaries show the existence of a monomer-like emission exclusively localized at the boundary. Using this dual PL/LD microscopy setup, we conducted a systematic survey of grain boundaries and established a dependence of the localized luminescence intensity on the relative orientation of exciton dipoles at the boundary. These observations indicate the grain boundaries are not molecularly abrupt but rather disordered regions of finite width. We also established that this localized state can be eliminated in films with large long grains where the relative angle between crystalline axes in adjacent grains is <5°.

## Results

### Soluble phthalocyanine: a ubiquitous molecule revisited

**Figure 1:**
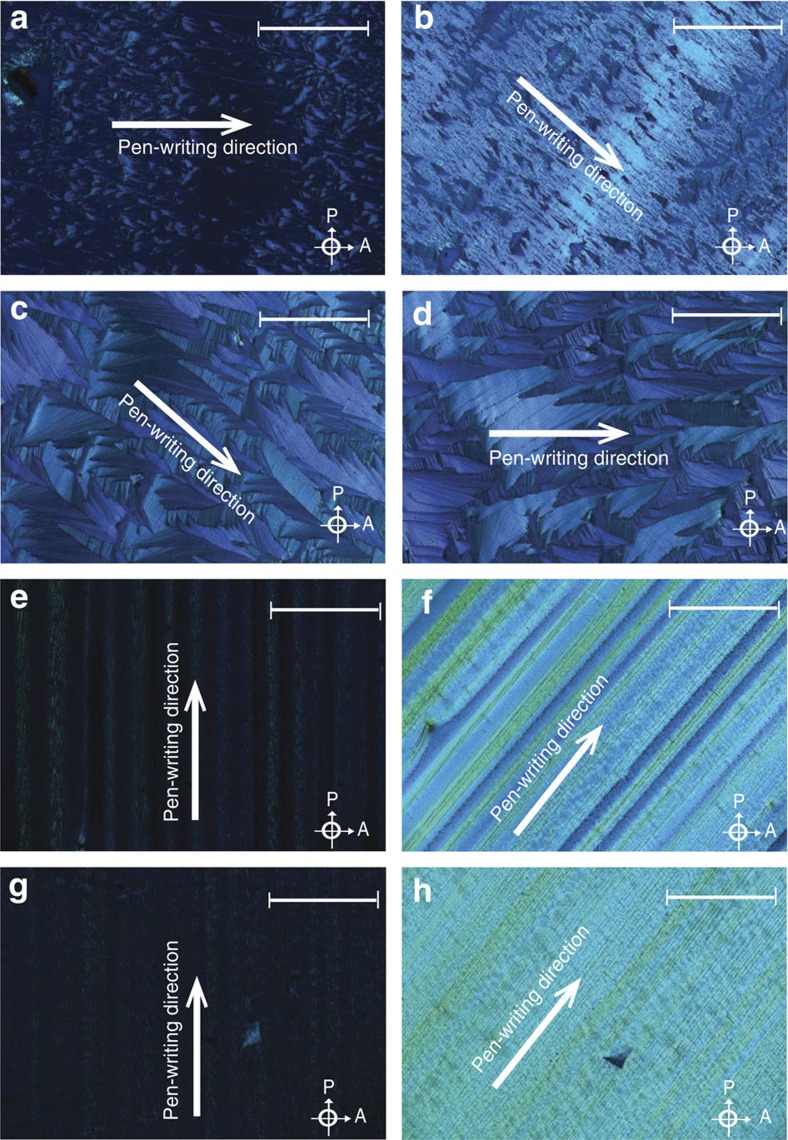
Long-range ordering in phthalocyanine thin films. Polarized microscopy images of crystalline H_2_Pc-OC_8_ thin films deposited on *c* plane sapphire using the solution-processing technique. Optimization of deposition speed, solution concentration and choice of solvent, results in millimetre-sized grains suitable for optical spectroscopy experiments. (**a**,**b**) 0.5%, 11 μm s^−1^, toluene; (**c**,**d**) 1%, 18 μm s^−1^, toluene; (**e**,**f**) 1%, 40 μm s^−1^, chloroform and (**g**,**h**) 1%, 30 μm s^−1^, chloroform. During film deposition, the sample stage is moving along the direction marked with a white arrow in each image. The grains with molecular stacking axis oriented along the polarizer direction appear dark in these images. Scale bar, 500 μm.

Figure 1 displays standard polarized optical microscope images of four thin films of 2,3,9,10,16,17,23,24-Octakis(octyloxy)-29H,31-phthalocyanine (H_2_Pc-OC_8_), a commercially available peripherally substituted phthalocyanine (Pc) soluble in common organic solvents, our molecule of choice for the present study. The films were deposited at different writing speeds from chloroform or toluene solutions of various concentrations using a ‘pen-writing', hollow capillary technique^11^,^13^. In essence, this technique involves translating a 500-μm-wide glass capillary loaded with solution at a constant speed across a substrate. Optimal results are obtained when crystallization takes place at the contact line between the meniscus and the substrate. In all instances, we used crystalline *c* plane sapphire as substrate; however, similar results were obtained for other dielectric transparent substrates such as plain microscope glass or indium tin oxide. To produce films with macroscopic grain sizes, the capillary translation speed, the concentration of molecules in solution, and the choice of solvent must be optimized. The four films in Fig. 1 illustrate this optimization process that can be quite different for each molecule. In the case of H_2_Pc-OC_8_, the largest grain sizes and most uniform films were obtained when 1% chloroform solution was used (Fig. 1e–h). At the optimum deposition speed of 30 μm s^−1^ in chloroform (Fig. 1g,h), the grain size exceeds the microscope field of view. For toluene solutions, at lower concentrations (Fig. 1a,b), the optimized deposition results in a disordered film with small grains and poor substrate coverage, whereas for deposition speeds significantly larger than optimal the nucleation happens randomly and simultaneously over the entire area of the sample resulting in a ‘fan-like' polycrystalline structure (Fig. 1c,d). This latter case is quite an interesting one since it represents a situation reminiscent of the vapour-deposited films that are polycrystalline structures with randomly oriented grains. The fan-like structure can thus be regarded as a ‘scaled-up' model for the vapour-deposited films, with grain sizes large enough for optical spectroscopy investigations. For this reason, our studies of correlations between different grain boundaries and the nature of excited states at the boundary focused precisely on these types of disordered samples rather than the long-grain well-ordered structures that we explored elsewhere^14^.

**Figure 2:**
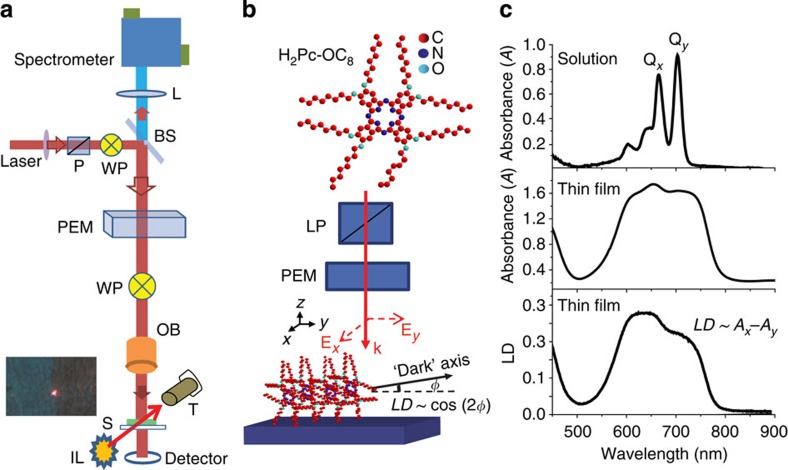
Linear dichroism (LD) and molecular ordering. (**a**) Schematics of the LD/PL microscopy experiment employed to study the relative orientation of crystalline grains in the H_2_Pc-OC_8_ thin films. The LD image is acquired in transmission geometry by scanning a polarization-modulated focused laser beam onto the sample surface while spatially- and polarization-resolved PL spectra are recorded in backscattering geometry, after removing the piezoelectric modulator and waveplate from the setup. BS, beam splitter; IL, illumination lamp; L, singlet lens; OB, objective lens; P, linear polarizer; PEM, piezoelectric modulator; S, sample; T, long working distance observation telescope; WP, half-wave plate. (**b**) Molecular structure of H_2_Pc-OC_8_ and the orientation of light polarization with respect to the ‘edge-on' molecular stacking. Light polarized perpendicular to the stacking (‘dark') axis is predominantly absorbed. (**c**) Typical absorbance (*A ∼αd*) and linear dichroism (LD *∼Ax−Ay*) spectra from an H_2_Pc-OC_8_ crystalline film deposited using the pen-writing technique (a reference spectrum of the same molecule in solution is shown for comparison).

Phthalocyanine, in its non-soluble form, is a well-studied ubiquitous molecule characterized by D_2h_ symmetry that results in optical transition dipoles polarized in the molecular plane. This symmetry and selection rules are preserved for the soluble, substituted Pc, whose absorbance spectrum measured in chloroform exhibits a splitting of the peak associated with the Q-band transitions (Fig. 2c)^15^,^16^,^17^. The optical dipoles associated with these transitions lie along mutually perpendicular directions in the plane of the molecule^18^,^19^ and photons absorbed or emitted are polarized in the molecular plane. The difference in absorbance (*A*_*x*_−*A*_*y*_) for light polarized parallel and perpendicular to the molecular plane is referred to as LD^20^. In solution or amorphous films, there is no preferential orientation of the molecular plane; therefore, no LD is observed in such cases. It is expected, however, that in a single crystal the long-range molecular order preserves the selection rules, hence the LD can be observed on a macroscopic scale^21^.

Out-of-plane X-ray diffraction (Supplementary Fig. 1) experiments we conducted at the synchrotron facility of Brookhaven National Laboratory confirmed our films crystallize in the orthorhombic phase with the molecules stacked edge-on to the substrate and the stacking axis parallel to the substrate. That means an experimental geometry, such as the one sketched in Fig. 2a,b, where the **k** vector of the incident light is perpendicular to the stacking axis, should render a significant amount of LD (LD *∼A*_*x*_*−A*_*y*_) provided the orientation of the molecular stacking remains the same at least on the length scale of the beam diameter. This orientation also explains the polarization-mode contrast observed in Fig. 1: if the stacking axis happens to be oriented along the polarizer axis the grain appears dark in the microscope image.

Typical absorbance (*A*) and LD spectra from a 0.1% film (Fig. 1c) show a large spectral broadening as a result of molecular interactions^22^,^23^,^24^, and exciton–vibrational coupling^17^,^25^,^26^,^27^. The LD spectrum is of a similar shape to the absorption and the large LD signal (about 30% of that of a commercial polarizer) observed for the bandgap spectral region, implies the majority of the transition dipoles are linearly polarized and oriented perpendicular to the pen-writing direction. Assuming molecular selection rules are preserved in the solid film, the large positive LD confirms the presence of long-range order, where most molecules stack face-to-face, 'edge-on', as illustrated in Fig. 2b. While the orientation of stacking axis can be inferred from the measured LD, it is important to emphasize that, unlike electron microscopy or X-ray diffraction which offer direct structural information but cannot probe extended excitonic states in crystals, optical spectroscopy techniques such as LD or PL microscopy selectively probe the electronic/excitonic band states and the optical transition dipoles orientations. Other absorbance spectra from the samples investigated in this work can be found in Supplementary Fig. 2, which indicates that there are no significant changes in the spectral shape. The overall absorbance does scale with film thickness as expected. All subsequent film thicknesses mentioned in the present work are estimated based on a calibration of the deposition system presented in the Supplementary Information of ref. 12.

### Electronic states localized on grain boundaries

The influence of molecular ordering on excitonic states, radiative recombination and states originating at the grain boundaries is probed when spatially resolved PL and LD are simultaneously recorded from a selected area using a focused narrowband laser probe beam. To this end, we combined LD and PL scanning microscopy as illustrated in Fig. 2a to unambiguously establish a particular grain orientation, while selectively probing the bandgap luminescence with a spatial resolution of ∼5 μm. A detailed description of the experimental setup is also available in the Methods section of this paper.

**Figure 3:**
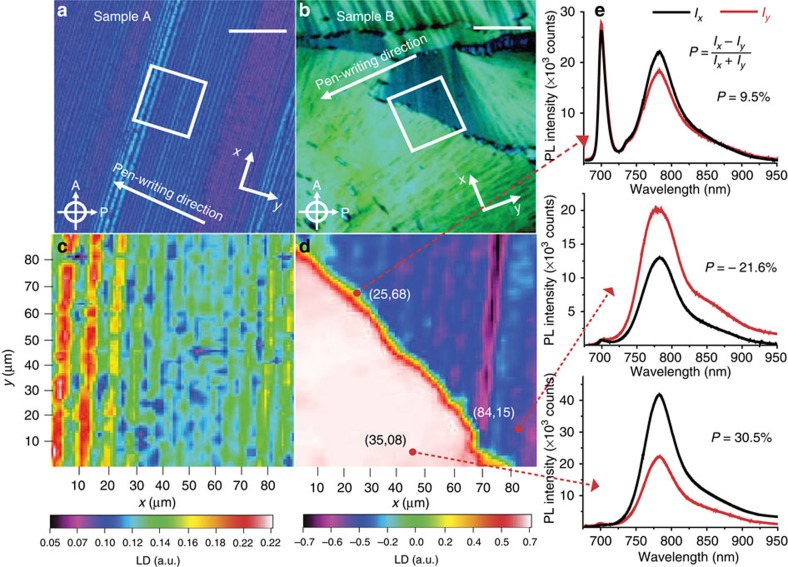
LD/PL microscopy of electronic states at the grain boundaries. (**a**,**b**) Standard polarization-mode microscopy images of two distinct polycrystalline H_2_Pc-OC_8_ films: sample A (0.1% solution, 2 mm s^−1^) and sample B (1% solution, 20 μm s^−1^) that reveal very different grain structures. The orientation of pen-writing direction with respect to the polarizer and analyser (*x–y*) axes is marked with a white arrow. (**c**,**d**) High-resolution LD microscopy images of 90 × 90-μm areas identified with a white square in (**a**,**b**). (**e**) polarization and spatial resolved PL spectra of sample B recorded at three distinct locations identified through their (*x*,*y*) coordinates. The LD contrast indicates the different orientation of the stacking axis in adjacent grains. Depending on the growth conditions, the grains assemble in stripe-like films with low-angle (∼5°) grain boundaries (sample A (**a**,**c**)) or fan-like films with high-angle (∼25°) grain boundaries (sample B (**b**,**d**)). An additional monomer-like feature is present at the high-angle grain boundary. Similar spectra are recorded from sample A, but no monomer-like feature is observed at the low-angle grain boundaries.

The three samples we explored with our scanning microscopy experiment are presented in Fig. 3a,b and Supplementary Fig. 3. The 30-nm-thick sample A (fabricated from a chloroform solution of 0.1% at a pen-writing speed of 2 mm s^−1^) exhibits a stripe-like geometry where all molecules within any individual stripe are oriented in the same direction, with some difference in orientation between two adjacent stripes, which accounts for the image contrast. For larger deposition concentrations, the microscope image of the 200-nm-thick sample B (deposited from a chloroform 1% solution at 20 μm s^−1^ pen-writing speed) reveals more randomly oriented grains with ‘fan-like' structures that are expected in a concentrated solution with many nucleation centres. All films have grain sizes ranging from 10 to 100 μm, enabling us to spatially resolve the properties of electronic states in the vicinity of the bandgap within a single-grain and probe single-grain boundaries using this dual-microscopy technique.

Figure 3c,d reproduces LD two-dimensional (2D) scans of 90 × 90-μm areas marked with white squares in the microscopy images (Fig. 3a,b). The pen-writing direction for each sample is also indicated in the microscope image. Sample A exhibits parallel stripes of ∼10 μm in width, orthogonal to the pen-writing direction. The LD values are positive across the scanned area with a 25% difference between adjacent stripes. Since the LD is proportional to cos (2*ϕ*)^21^, where *ϕ* is the angle that defines the stacking axis orientation with respect to the polarizer axis (Fig. 2), the LD contrast observed in sample A corresponds to a relative angle Δ*ϕ* ∼5^0^ between stacking axes from adjacent stripes. In striking contrast, sample B exhibits a distinctly different, ‘fan-like' structure with well-defined grain boundaries in polarization-mode microscopy images. The LD scan across an area traversed by a single-grain boundary, revealed a very large contrast between the adjacent grains corresponding to a relative angle Δ*ϕ* ∼25^0^ between the stacking axes of the two grains. A preliminary LD imaging survey conducted on films deposited at different concentrations ranging between 0.1% and 1% indicated that there is a direct relationship between the relative orientation of the stacking axes at the grain boundaries and solution concentration and deposition speed. A film of intermediate concentration and thickness (0.5%, 150 nm) deposited with the same writing speed (20 μm s^−1^), sample C, has parallel grains with large positive LD. The sample thicknesses quote here were estimated based on the thickness versus deposition speed curve found by Cour *et al.*^12^ The absorbance measurements performed on our samples (Supplementary Fig. 2) are consistent with this estimation.

All our films exhibit strong room temperature PL resonant to the absorption edge. Since radiative transitions typically originate from the lowest vibrational state of an electronic excited state^26^, the absence of a large Stokes shift is indicative of strong exciton vibronic coupling and the existence of delocalized excitons^26^. This is a consequence of the long-range ordering present in our films, that is, the size of crystalline grains, is significantly larger than the typical exciton diffusion lengths^9^, such that excitons generated inside a grain recombine before reaching the grain boundary or a defect state.

Figure 3e highlights the most interesting results of the luminescence microscopy experiments. First, the luminescence is linearly polarized perpendicular to the stacking axis, indicating that the room temperature emission is indeed associated with the recombination of the singlet exciton ground state. The π-orbital overlap between adjacent molecules preserves to some extent the monomer selection rules for the ground-state transition dipole at room temperature. In Fig. 3 micro-PL spectra recorded at coordinates (*x*,*y*)=(35,08) and (84,15) indicate the luminescence polarization is correlated to the stacking axis orientation, changing sign and magnitude in tandem with the LD. The delocalization of this bandgap singlet exciton state has been described in detailed elsewhere^14^. Second, in addition to the expected singlet exciton recombination feature, the emission spectrum recorded from the grain boundary (for example, (*x*,*y*)=(25,68)), exhibits an additional strong sharp feature centred at 700 nm. This feature is exclusively observed when the laser excitation beam is focused anywhere along the grain boundary.

**Figure 4:**
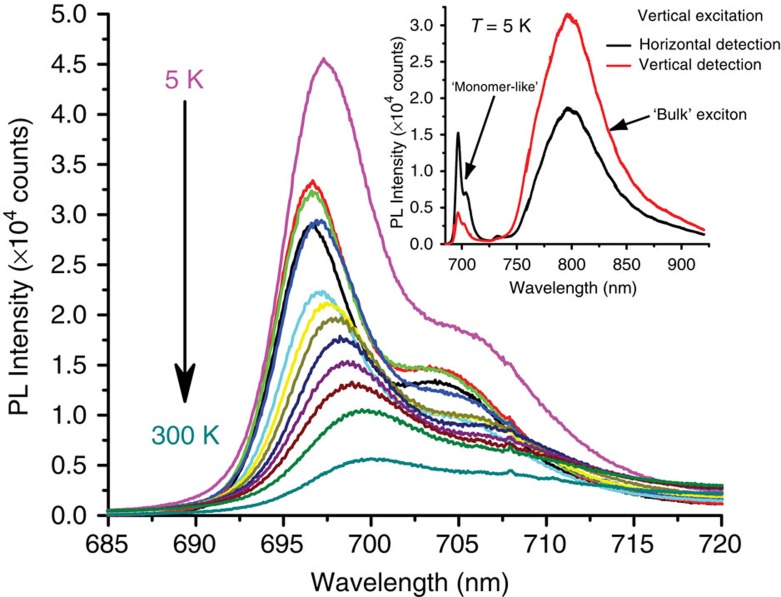
Temperature evolution of luminescence from grain boundaries. PL spectra from the grain boundary in Fig. 3d recorded at various temperatures between 5 and 300 K. The sharp, ‘monomer-like' feature exhibits the typical evolution of radiative emission from highly localized electronic states: the spectrum redshifts and broadens with increasing temperature. The inset displays both *x*- and *y*-polarized components of the luminescence spectrum indicating the localized, grain boundary emission and the ‘bulk' photoluminescence have opposite polarizations.

**Figure 5:**
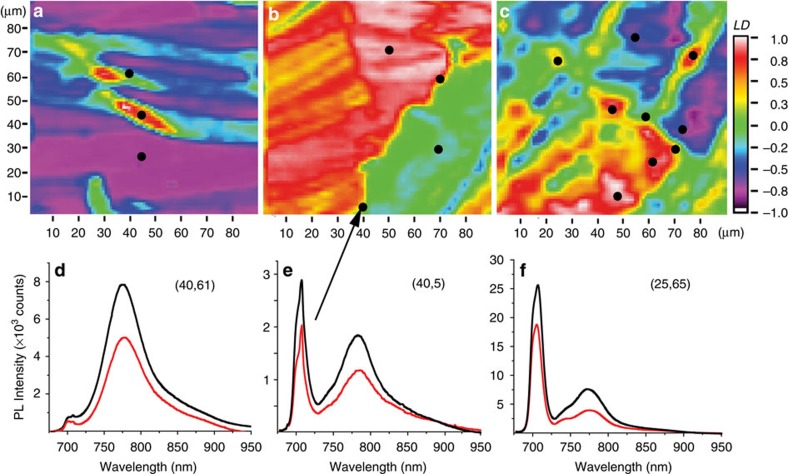
LD/PL microscopy survey of grain boundaries. (**a**–**c**) LD microscopy images of individual grain boundaries from samples fabricated at different concentrations and writing speeds. The linear dichroism is normalized to the absorbance, for a direct comparison between samples (**a**,**b**) 0.5%, 30 μm s^−1^ and (**c**) 0.5%, 20 μm s^−1^. (**d**–**f**) Polarization and spatial resolved photoluminescence spectra recorded at locations marked with black dots in (**a**–**c**), respectively. The beam locations are identified through their (*x*,*y*) coordinates. The correlation between relative grain orientation and localized emission intensity is summarized in Table 1.

To investigate its origins, we placed the sample in a variable temperature liquid He continuous flow cryostat and recorded PL spectra from grain boundaries at various temperatures ranging from 5 to 300 K (Fig. 4). The sharp PL feature redshifts and decreases in intensity, reminiscent of the behaviour of typical emission from localized states in semiconductors. Its polarization at low temperatures (Fig. 4 inset) was opposite to that of the bandgap exciton recombination. Following this observation, we conducted a systematic PL/LD study of grain boundaries in films deposited under different conditions, surveying ∼20 grain boundaries. Images from three representative grain boundaries and the luminescence spectra associated with them are shown in Fig. 5. The first observation is that the relative intensity of the grain boundary luminescence that we define as ***I***_**relative**_=***I***_**monomer**_/***I***_**total**_ varies greatly from one-grain boundary to another and remains approximately constant along the grain boundary. The second observation is the existence of a correlation between this relative intensity and the relative orientation of adjacent grains. This correlation is readily apparent from the data summarized in Table 1 which indicates that the monomer-like emission intensity increases with increasing boundary angle, reaching a maximum in the more disordered samples where we can no longer speak of a boundary between two crystalline grains but rather of disordered areas with sizes comparable to the crystalline regions (Fig. 5c). In our experiments, these disordered areas are unambiguously distinguished from crystalline grains oriented at 22.5^o^ because they exhibit LD=0 regardless of the incident light polarization

## Discussion

In spite of our study being focused on a specific soluble derivative of phthalocyanine, it is very useful to consider the results of our LD imaging and spatial- and polarization-resolved PL studies in light of the knowledge they bring on the correlations between electronic states and molecular ordering for small molecules systems in general. In recent years, numerous fundamental and applied studies of electron mobilities and exciton diffusion on small-molecule organic semiconductor thin films such as pentacene, rubrene and even phthalocyanines report ever increasing record mobilities and diffusion lengths, far exceeding early predictions that follow from a simple ‘oriented gas' model^17^. In all cases, the key factor seems to be the reduction and control of grain boundaries such that continuous improvement in the thin film quality should reveal exciting new physics and bring a better understanding of electronic phenomena in these materials, in the same way that molecular beam epitaxial growth led to very high mobilities and excitons diffusion lengths in inorganic semiconductors.

These materials seem to belong to an intermediate regime where the delocalized carriers and excitons are neither completely localized like the Frenkel excitons nor delocalized like the Wannier excitons in inorganic semiconductors^14^,^28^. In this context, optical spectroscopy techniques are ideal because they probe the longer-range interactions and excitations while providing spatial resolution on the micron range, which is now sufficient given the increasingly large crystalline grain size. The scanning narrowband laser microscopy we employed here is a powerful technique that has been used to explore optical properties in a variety of systems. Its different flavours (Kerr Rotation, Faraday rotation, MCD, or LD) are employed for studying a vast array of phenomena ranging from spin-polarized electron transport in GaAs to grain boundaries in halide systems^29^,^30^.

Phthalocyanines are the ideal test system for such investigations because they are resistant to oxidation and retain a high yield of radiative recombination in crystalline films. Most of them are readily available and, as mentioned earlier, very well studied as far as the molecular properties are concerned. Moreover, the success we had in depositing thin films with macroscopic order (Fig. 1) proved the robustness of this technique that relies on nucleation and surface tension control, a technique originally designed and optimized for depositing pentacene thin films^13^. (Our group ultimately obtained macroscopic grain sizes for two distinct substituted metal phthalocyanines as well as two additional molecules in the porphyrin family: tetra-phenyl porphyrin (TPP) and naphthalocyanine (NPc). Optical studies on these species that investigated different aspects of longer-range interactions are beyond the scope of the present paper and will be reported elsewhere).

The necessity of an optical spectroscopy spatially resolved study stems from the progress made in fabricating OFET-like structures that investigate the influence of single-grain boundaries on electron mobilities^6^,^11^,^12^,^31^. These studies looked into how the nature and number of grain boundaries limit the ultimate mobility through the presence of localized states (traps) at the boundary. They also established the nature of grain boundaries in the thin films deposited through all flavours of solution-processing techniques is very different from that of grain boundaries in vapour-deposited thin films, a difference that originates in the different molecular ordering along the high-mobility axis (*π*-stacking versus herringbone). Specifically, transport measurements indicated the grains are separated by disordered regions of finite thickness (of the order of 10 nm) rather than molecularly abrupt boundaries^11^. Very recent spatially resolved transient absorption measurements on soluble pentacene thin films also indicate the boundaries should be viewed as disordered regions^32^. In this context, the observation of radiative emission localized at the grain boundary (Figs 3 and 5) is somewhat expected and must be associated with these disordered regions. The unexpected element is the wavelength (or energy) associated with this emission, 700 nm (or 1.77 eV), significantly larger than the bulk luminescence located at 785 nm (∼1.58 eV). In a simplistic approach, we can interpret the 200 meV difference as a quantitative estimate for an energy barrier the excitons/carriers need to overcome to reach the adjacent grain boundary. Scattering from this 200 meV barrier will represent one of the mechanisms limiting the diffusion length/mobility, in addition to trap states present at the boundaries. Imaging of different films and grain boundaries such as the ones presented in Fig. 5 allow us to hypothesize on the nature of electronic states at the boundary and refine this energy barrier concept.

For most films, the grain boundary is certainly narrower than our laser beam diameter (∼5 μm), that explains the bulk exciton contribution to the PL spectra. In the more disordered regions such as the one probed in Fig. 5c the grain boundaries seem to have evolved into macroscopic disordered regions (LD=0) that are certainly in the micron range. These are the very same regions where the sharp 700 nm PL feature becomes the dominant feature in the spectrum (Fig. 5c). In addition, our grain boundary survey summarized in Table 1 indicates that the relative intensity of this boundary feature increases with increasing angle Δ*ϕ* between stacking axes of adjacent grains. It is also important to note that there are preferred recurrent relative angle orientations, listed in Table 1. In spite of surveying numerous grain boundaries, these were the only orientations we found within an uncertainty of ∼2°.

On the basis of these results, we propose that the grain boundaries are indeed disordered regions (as previously inferred by transport studies) where we observe a PL spectrum reminiscent of a solution spectrum or weakly interacting molecules with reduced orbital overlap. The relatively large polarization observed at low temperatures (Fig. 4 inset) is not a signature of a selection rule at the grain boundary but rather a consequence of the absorption and emission selection rules for the bulk exciton originating from the crystalline regions immediately adjacent to the grain boundary^14^.

Finally, we point out the sharp luminescence feature completely disappears in the highly ordered films such as the ones in Fig. 1g,h. We expect the nature of grain boundaries to be entirely different in these films because they are deposited in what has been identified as the convective regime^12^, that is, the liquid transforms into a solid film at a well-defined boundary that is <3-μm thick. Studies that investigated correlations between grain boundaries and electronic mobilities^29^ indicate that not only is the mobility greatly enhanced when the number of grain boundaries the electron cross is reduced, but more importantly, the mobility is significantly increased in films deposited in the convective regime. Our optical spectroscopy results now provide a quantitative feedback on the bandgap electronic states at the grain boundaries. On the basis of the observations of localized emission, we propose that the electron mobility as well as exciton diffusion will also be reduced in these systems because of the lack of ordering and poor orbital overlap at the grain boundary, even at high excitation rates where trap states are saturated. While lower energy trap states are not luminescent, their presence can be inferred from the evolution of the PL intensity with excitation power shown in the Supplementary Fig. 4b. At low power, the PL intensity is proportional to *N*^0.8^, where *N* represents the number of incident excitation photons, suggesting the presence of a non-radiative relaxation mechanism for excited carriers that may involve low-energy traps. This mechanism is most likely the Shockley–Read–Hall recombination, previously identified in solution-processed thin films^33^. For excitation densities larger than 10^25^ photons per second per centimetre^3^, the emission intensity increases as the square root of excitation power, a possible signature of the Auger exciton–exciton annihilation at large excitation powers^34^. In these films, the observation of the sharp luminescence feature is independent of the nature of non-radiative traps or the complex nature of non-radiative quenching mechanisms, because the spectral shape and relative PL intensity does not change with excitation power as evidenced by Supplementary Fig. 4a. Moreover, absorbance spectra measurements, PL power studies or PL lifetime measurements (Supplementary Figs 2, 4 and 5) indicate the two transitions originate from entirely spatially separated states that do not interact with each other through energy transfer mechanisms. PL decays for both features are characterized by a single exponential and the same lifetime, in contrast to systems where the presence of energy transfer shortens the higher energy feature lifetime^35^. Parameters such as film thickness or excitation power only seem to affect the overall PL intensity for both features.

In conclusion, the adaptation of polarization-resolved laser scanning techniques to crystalline organic semiconductor thin films have the potential for revealing a wealth of information in regards to the nature of excited states in these systems, with emphasis on longer-range phenomena. Using the LD/PL scanning microscopy in tandem with novel thin film deposition techniques such as the hollow capillary pen-writing technique that relies on careful control of nucleation, we were able to unveil the nature of excited states at a single-grain boundary in phthalocyanine thin films. Specifically, we concluded that scattering from an energy barrier created in a disordered region is one of the mechanisms limiting exciton diffusion length in the presence of grain boundaries, in addition to the low-energy traps. In a broader sense, the macroscopic grain sizes open up the organic semiconductor systems for the first time to the wealth of bandgap spectroscopy experiments that can investigate the nature of excited states and the correlations between the crystalline structure and these excited states in a sample free of grain boundaries or defects. For example, our group is making strides towards understanding the bandgap luminescence and the spatial extent of the bulk exciton wavefunction in these films some of which were recently reported elsewhere^14^.

## Methods

### Materials and thin film fabrication

The dye molecules of 2,3,9,10,16,17,23,24-Octakis(octyloxy)-29H,31H-phthalocyanine (H_2_Pc-OC_8_) were purchased from Sigma Aldrich. The materials were purified by column chromatography using chloroform as an eluent. Thin films were deposited on substrates by a solution processing pen-writing technique at room temperature using either toluene or chloroform as the solvent^13^. This method employs a hollow borosilicate glass capillary pen and solutions with concentration of 0.1–1 wt% are held in the pen by capillary forces. Film deposition is accomplished by allowing droplets of solution at the end of the pen to make contact with the substrate and then laterally translating the pen at a controlled speed ranging between 0.02 and 2 mm s^−1^. The deposition, conducted at room temperature, produces highly ordered films with uniform thicknesses ranging between 20 nm and 1 μm depending on the deposition parameters. The substrates were thoroughly cleaned and treated with methanol to improve the wettability and to assist with forming uniform films.

### LD and PL experiments

The absorption and LD spectra were measured at room temperature in the range of 400–1,100 nm using a quasi-monochromatic (1-nm bandwidth), tuneable, incoherent, tungsten halogen monochromator light source. A continuous wave (CW)-focused HeNe laser was employed as probe beam for LD microscopy measurements. The same laser served as quasi-resonant excitation for spatially resolved PL measurements (Fig. 2a). To record LD images of individual crystalline grains, we adapted a polarization-resolved microscopy technique formerly employed to image spin drift and diffusion in GaAs^29^. A piezoelastic modulator was placed in the beam path to modulate the light polarization from *x* to *y* polarized at a frequency of 100 kHz. The laser beam was focused to a ∼5-μm spot using a × 10 objective lens mounted on a piezo nanopositioning stage. A long working distance (15 mm) objective lens was chosen to insure that the experiment can be performed at temperatures as low as 5 K in an existing Oxford cryostat. These low-temperature experiments were reported elsewhere^14^. The 2D LD images were obtained by raster scanning the focusing objective lens. Spatially resolved PL was simultaneously collected with the same lens in backscattering geometry and spectrally resolved using an Acton spectrometer coupled with a Roper Scientific liquid nitrogen cooled charge-coupled device. PL decay times presented in the Supplementary Information section were measured using the 2 ps, 86 MHz, 400 nm frequency doubled output of a Ti-Sapphire oscillator as excitation and a PicoQuant Time Correlated Single Photon Counting System with an Instrument Response Function (IRF) of 120ps connected to a PMT detector mounted on the side port of the Acton Spectrometer. All PL/LD experiments were carried out at low excitation powers (<100 μW) to avoid exciton–exciton annihilation or exciton–polaron interactions (Supplementary Fig. 4). For the PL decay measurements presented in Supplementary Fig. 5, we estimated the number of photons per pulse absorbed by the films is ∼3 × 10^6^, while the number of molecules illuminated, estimated from the film crystal structure and the beam size, is a thousand times larger. All spectra were corrected for the charge-coupled device response and grating reflectivity across the spectral range of interest. To compensate for the difference in grating reflectivity for *s-* and *p-*polarized light, a quarter waveplate was inserted between the PL polarization analyser and the spectrometer slit. A correction factor was also introduced to account for the differences in the *s* and *p* reflectivity and transmittance of the beam splitter used in these experiments. Temperature-dependent PL studies were conducted in a continuous flow Oxford Instruments optical cryostat where the sample temperature was continuously varied from 5 to 300 K.

### X-ray diffraction

We independently confirmed the ‘edge-on' stacking through out-of-plane X-ray scattering studies performed at the NSLS-I facility of the Brookhaven National Laboratory. The 2D scattering map for sample A, shown in Supplementary Fig. 1, reveals clear peaks at *2θ*=3°, 6° and 9°, corresponding to the (100) orthorhombic reflection with a lattice parameter **a**=23.7 Å. This number matches the lattice constant of α-phase H_2_Pc and confirms the in-plane orientation of the stacking axis. The *2θ*=3° region of the scattering map is re-plotted in Supplementary Fig. 1b on a logarithmic scale to highlight the arc-like diffuse scattering, which indicates the presence of some disorder in the film. A complete 3D structural determination is not yet available for H_2_Pc-OC_8_.

## 

## Additional information

**How to cite this article:** Pan, Z. *et al.* Polarization-resolved spectroscopy imaging of grain boundaries and optical excitations in crystalline organic thin films. *Nat. Commun.* 6:8201 doi: 10.1038/ncomms9201 (2015).

## Supplementary Material

Supplementary Information Supplementary Figures 1-5

## Figures and Tables

**Table 1:** Summary of grain boundary luminescence survey.

**Δ*****ϕ*** **(°)**	***I***_**relative**_**=*****I***_**monomer**_**/*****I***_**total**_
<5	0
11	0.07
14	0.33
22	0.63
27	0.35
Disordered film	0.8

Measurements of grain boundary luminescence intensity as a function of grains orientation reveal the ‘monomer-like' emission is only present at large angle Δ*ϕ*>5° grain boundaries. The largest intensities are measured in samples where crystalline grains are separated by disordered regions. The survey covered twenty distinct grain boundaries. Three of them were highlighted in Fig. 5.
