# Supplementary material for: Polarization-resolved spectroscopy imaging of grain boundaries and optical excitations in crystalline organic thin films
Source: Nat Commun. 2015 Sep 14;6:8201. doi: 10.1038/ncomms9201 (PMC4579592; doi:10.1038/ncomms9201)
Supplement: Supplementary Information — Supplementary Figures 1-5 [file ncomms9201-s1.pdf]

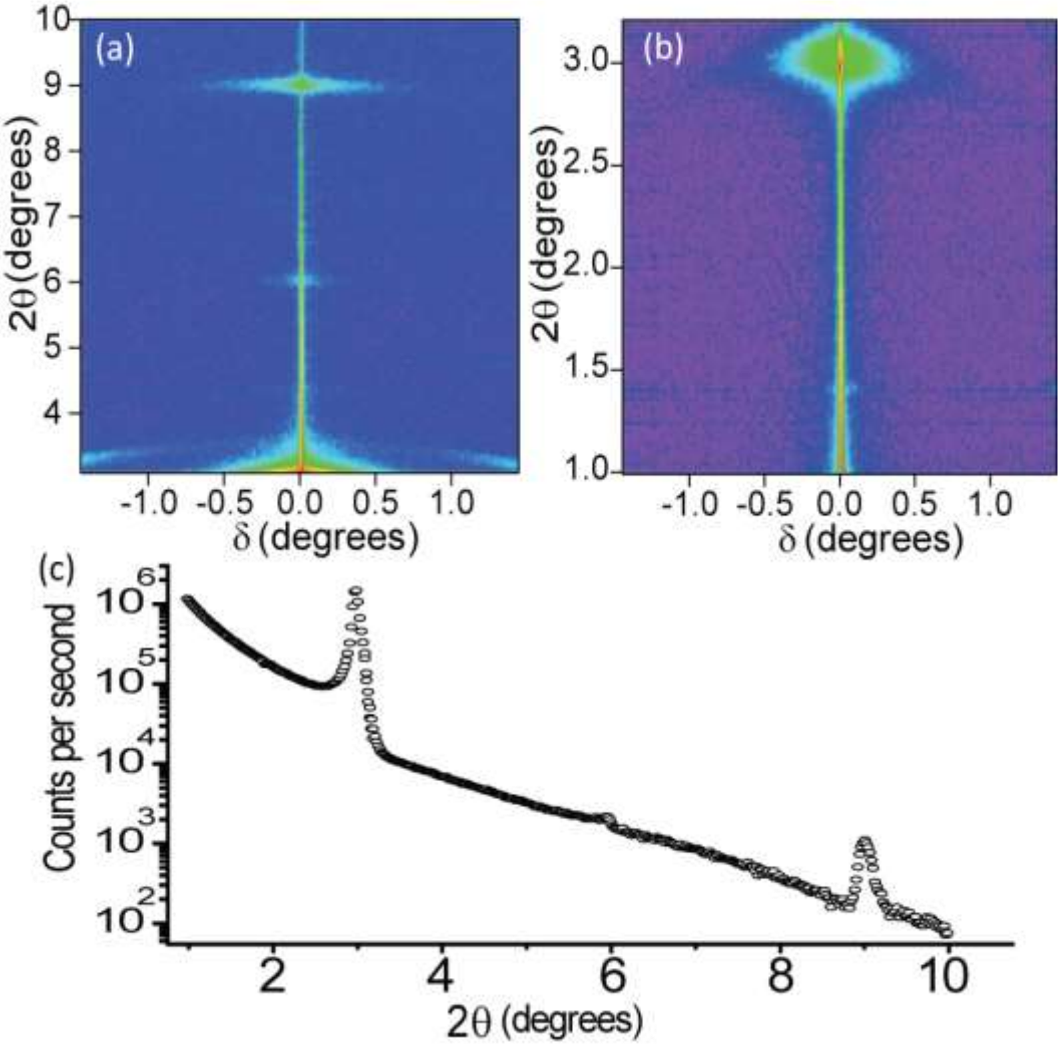

Supplementary Figure 1. X-Ray scattering (a) 2D x-ray scattering map of the (100) orthorhombic plane reflections in sample “A” at $2\theta = 3^0$, 6^0, and 9^0 degrees (b) $2\theta = 3^0$ diffraction pattern reveals disorder is present in the sample (c) 2θ XRD scan from sample A

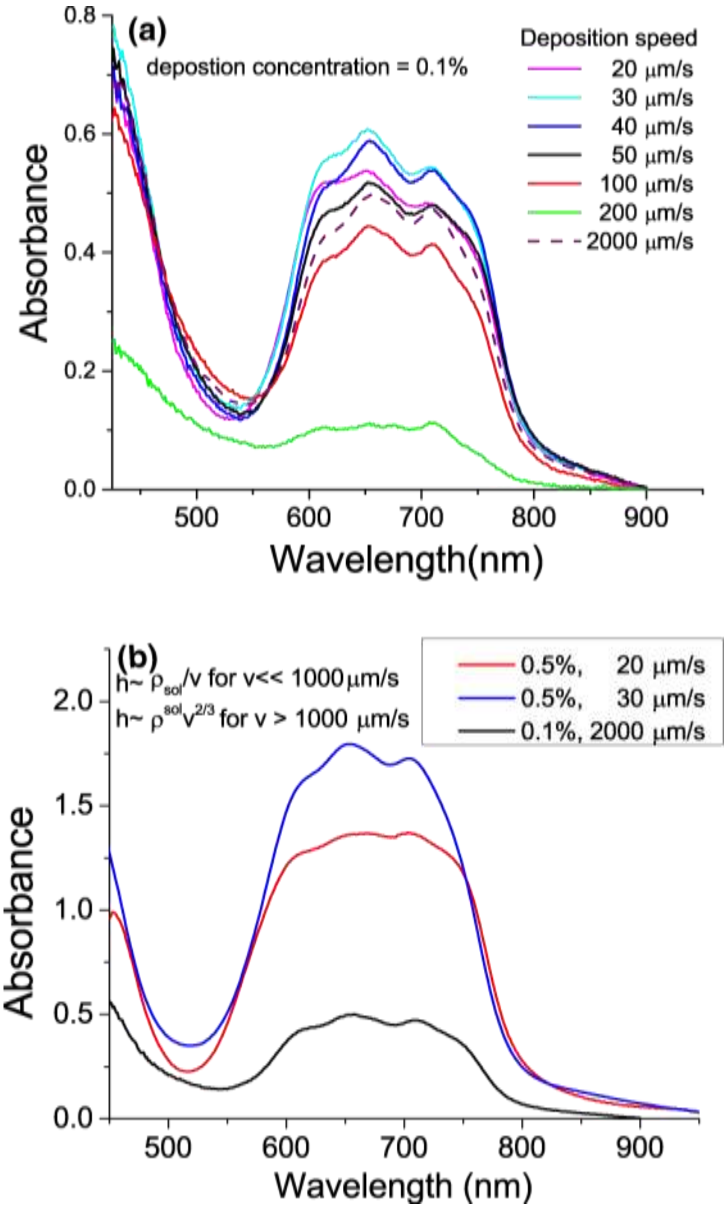

Supplementary Figure 2. Thin film absorbance (a) Evolution of absorbance spectra with film deposition speed for a series of $H_2Pc\text{-}OC_8$ films fabricated from the same 0.1% solution. The overall trend is in agreement with the prediction for the thickness dependence on deposition speed found in reference 12. In the case of the highest deposition velocity (2000 μm/s) the substrate coverage is very poor, resulting in a very small absorbance level. (b) Absorbance spectra recorded for the samples used in Figs 3 and 4 of the main text. The 0.5% samples were used in the grain boundary luminescence survey that generated Table I in the main text.

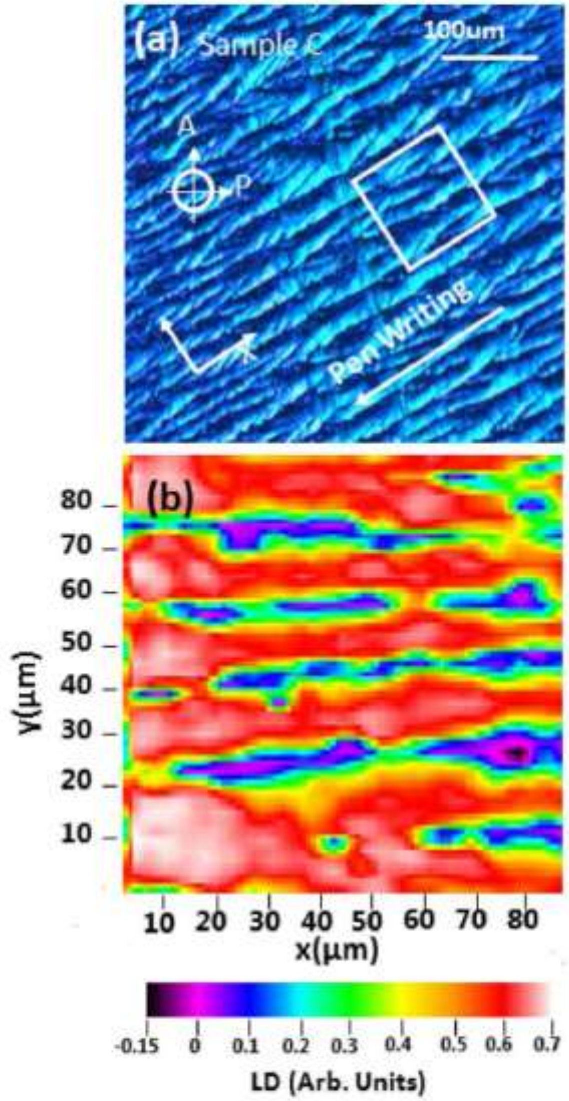

Supplementary Figure 3. LD microscopy from Sample C (a) Polarization mode microscope image of sample “C”. The orientation of pen-writing direction with respect to the polarizer and analyzer (*x*-*y*) axes is marked with a white arrow. The scale bar is 100 µm (b) High resolution LD microscopy image of a 90 X 90 µm area identified with a white square in part (a). The LD contrast indicates the different orientation of the c-axis in adjacent grains.

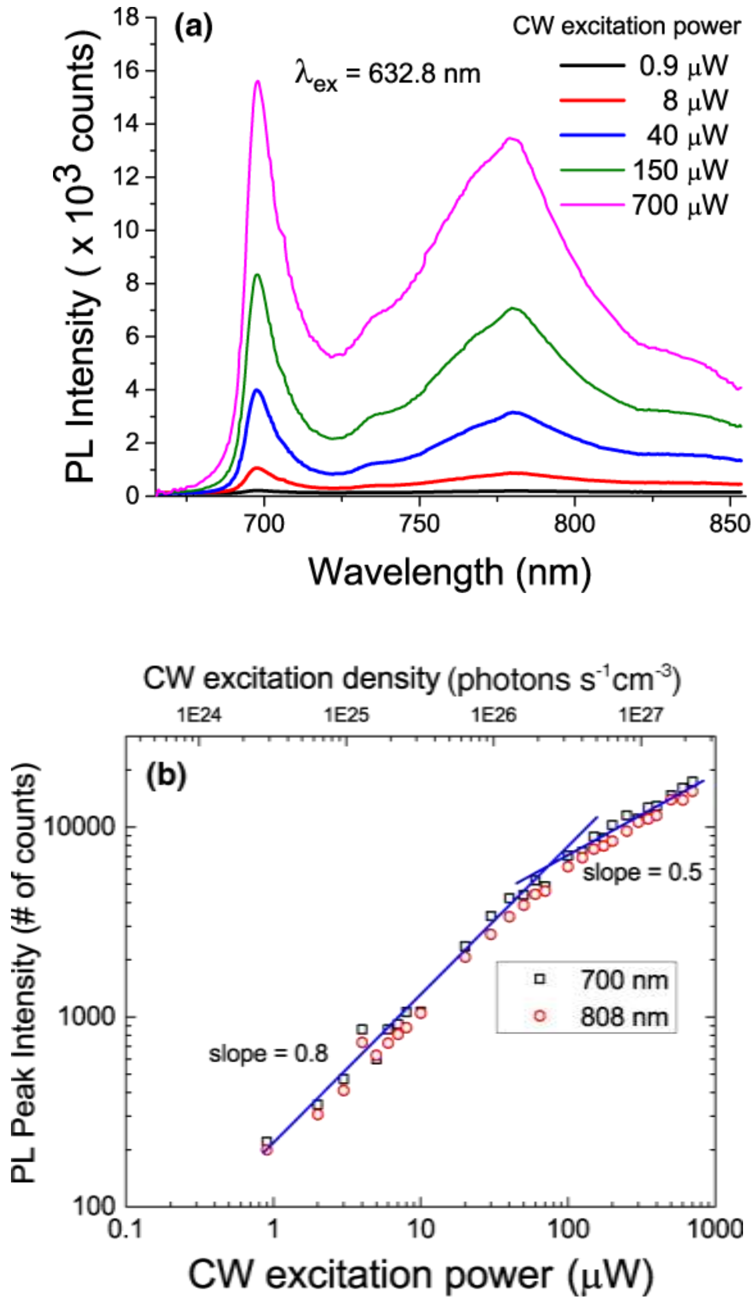

Supplementary Figure 4. Photoluminescence power study (a) CW photoluminescence spectra from Sample B recorded at different excitation powers using the PL/LD dual microscopy setup presented in the main text. The intensity ratio of the two luminescence features remains constant over the entire range of excitation powers. (b) PL intensity as a function of excitation power for the two features in the luminescence spectrum. At low powers the spectrum is dominated by single exciton recombination. A possible Auger exciton-exciton annihilation process is present for

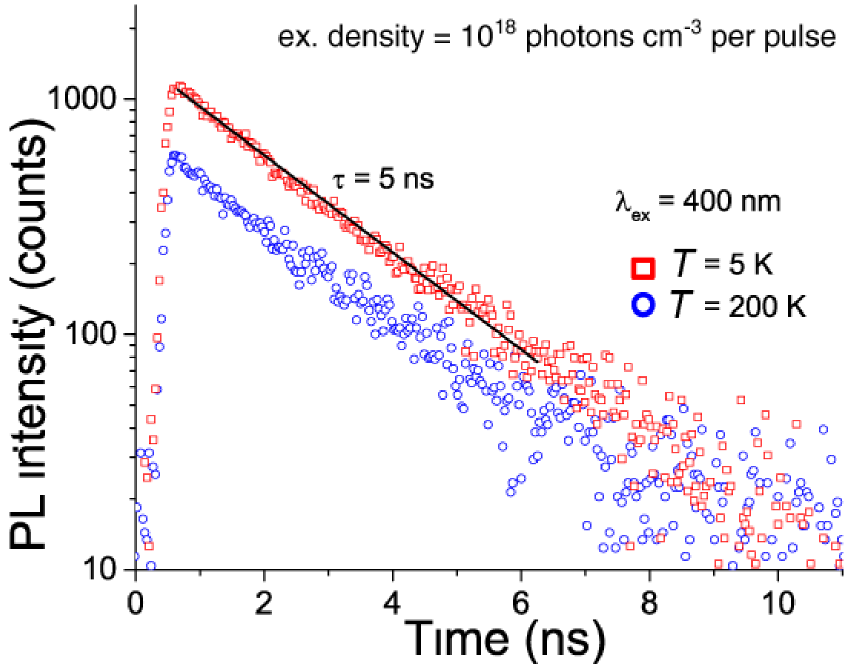

Supplementary Figure 5. Photoluminescence lifetimes. Photoluminescence decay recorded at 5K and 200K for the grain boundary feature (λ = 700nm). The measured decay lifetime equals 5 ns and is temperature independent. There is no indication of a temperature dependent energy transfer mechanism or non-
